# Optimization of the Simple One-Step Stool Processing Method to Diagnose Tuberculosis: Evaluation of Robustness and Stool Transport Conditions for Global Implementation

**DOI:** 10.1128/spectrum.01171-23

**Published:** 2023-06-26

**Authors:** Bazezew Yenew, Petra de Haas, Getu Diriba, Abebaw Kebede, Bihil Sherefdin, Yohannes Demissie, Ahmed Bedru, Mamush Sahile, Endale Mengesha, Zewdu Gashu Dememew, Ben Tegegn, Andrii Slyzkyi, Misikir Amare, Muluwork Getahun, Saro Abdella, Degu Jerene, Edine Tiemersma

**Affiliations:** a Ethiopian Public Health Institute, Addis Ababa, Ethiopia; b KNCV Tuberculosis Foundation, The Hague, The Netherlands; c Department of Microbial, Cellular and Molecular Biology, College of Natural and Computational Sciences, Addis Ababa University, Addis Ababa, Ethiopia; d Africa Centres for Disease Control and Prevention, Addis Ababa, Ethiopia; e KNCV Tuberculosis Foundation Ethiopia, Addis Ababa, Ethiopia; f Addis Ababa City Health Bureau, Addis Ababa, Ethiopia; Johns Hopkins University School of Medicine

**Keywords:** children, Ethiopia, robustness, simple one-step (SOS) stool method, stool, TB diagnosis, tuberculosis, Xpert MTB/RIF (Ultra) assay, storage conditions

## Abstract

Stool is recommended as an alternative specimen for the diagnosis of tuberculosis (TB) in young children, as they cannot easily produce sputum. The Simple One-Step (SOS) stool processing method is a new and simple stool processing method for the detection of Mycobacterium tuberculosis (MTB) using Xpert MTB/RIF Ultra (Xpert-Ultra). We determined the robustness of the SOS stool processing method and stool specimen transport conditions in participants with confirmed TB. We processed stool using the standard protocol after simulated “transport,” varying time, and temperature, and experimented with slightly modified processing steps. We included 2,963 Xpert-Ultra test results from 132 stool specimens of 47 TB participants, including 11 children aged <10 years. We compared Xpert-Ultra processing errors and MTB positivity rates between standard and modified procedures. Minor deviations from the standard SOS protocol did not significantly impact the Xpert-Ultra test outcomes. The rate of Xpert-Ultra processing errors significantly increased with noncold-chain transport, exposure of stool to sample reagent at room temperature or beyond 12 h, and adding >0.8 g of stool. We found that almost all steps in the current SOS stool processing method provide optimal Xpert-Ultra results but recommend an adjustment to use a wider range of stool amounts (0.3 to 0.8 g) than advised previously (0.8 g). With this adaptation, stool-based diagnosis of TB using the SOS stool processing method can be scaled-up.

**IMPORTANCE** The manuscript will support the global implementation and scale-up of the SOS stool method in routine settings. It also provides important insights on the optimal stool transport conditions and robustness of the SOS method, which can be used for bacteriological diagnosis of TB in children at the lowest levels of the healthcare system, avoiding lengthy healthcare-seeking pathways and additional costs.

## INTRODUCTION

Diagnosis of tuberculosis (TB) in young children remains a major global challenge primarily due to the paucibacillary presentation and difficulty in obtaining sputum specimens for diagnostic testing (1). Among the various specimen types recommended and being studied, stool is a noninvasive specimen with a high acceptability among caregivers compared to gastric aspirate or induced sputum (1, 2). In 2020, with an update in 2021, the World Health Organization (WHO) recommended Xpert MTB/RIF (Xpert) from a stool specimen as the initial test to detect MTB and its resistance to rifampicin (RIF) in children (3). In 2022, the more sensitive Xpert MTB/RIF Ultra (Xpert-Ultra) was introduced as a diagnostic tool for stool specimens (1). This guidance also provided recommendations on the stool processing methods to be used. Furthermore, the Global Laboratory Initiative (GLI) published a practical manual on stool testing in 2022, which provides practical guidance and considerations for the implementation of stool testing (4). A modeling study to measure the impact of implementing stool testing at the lower healthcare level showed an increase in bacteriological confirmation and a decrease in childhood mortality (5).

The SOS stool processing method, selected as one of the two methods recommended, is the easiest and most cost-effective method to implement in resource-limited settings (1, 4); it does not require supplies or equipment additional to what is needed for sputum Xpert and Xpert-Ultra testing. This method follows similar steps as sputum Xpert and Xpert-Ultra testing and is based on a single release-sedimentation step in which heavy fragments and debris settle by gravitation while bacteria remain in the supernatant during sedimentation (4, 6). The method showed similar sensitivity and specificity compared to other centrifuge-free methods tested in multiple head-to-head comparison studies (1, 6, 7). The method was also successfully introduced in routine settings in pilot studies in Addis Ababa (6) and Southwest Ethiopia (8) and in a nationwide pilot in Vietnam (9).

While the results showed reliable performance of the SOS stool processing method (1, 9), it remains essential to determine the tolerated deviation (robustness) of the method and to define whether optimization of critical steps in the protocol may increase its sensitivity and specificity and what conditions should be avoided to achieve optimal performance. It is also important to have more insight into the optimal stool transport conditions. As a result, a detailed study protocol was prepared describing experiments that will be performed using stool specimens collected from children and adults with bacteriologically confirmed TB in Addis Ababa, Ethiopia (10). The results of experiments (10) are summarized in this paper.

## RESULTS

**FIG 1:**
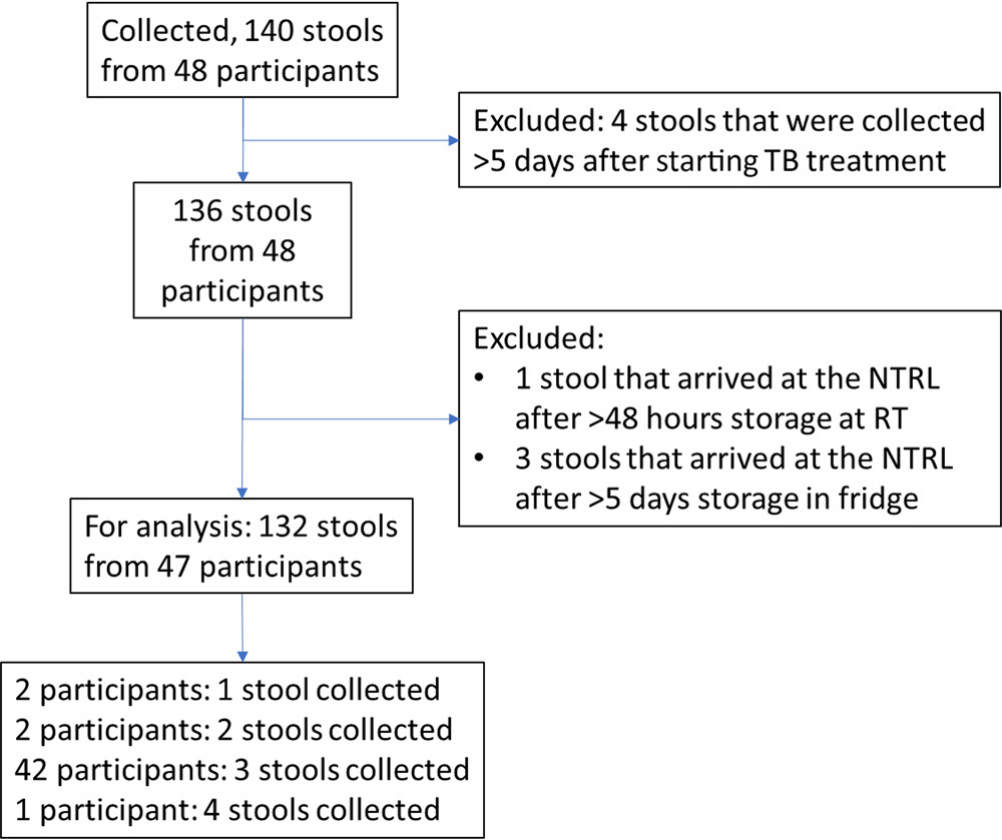
Flow diagram of the participants and stools collected. NTRL, National Tuberculosis Reference Laboratory; RT, room (or ambient) temperature.

A total of 48 participants were enrolled in this study, of whom 140 stools were collected. One participant and 8 stool specimens were excluded following the exclusion criteria, leaving 132 stools and 2,963 Xpert-Ultra test results for the final analysis from 47 participants. From most participants (42/47), three stool specimens were obtained (Fig. 1).

Of the 47 participants, 11 (23%) were children, and 36 (77%) were adults (Table 1). Most stool specimens (80%) were semiformed; only 1 liquid specimen (from an adult) was collected. Most specimens (92%) were collected on the spot, but for children, 26% of the specimens were collected at home (versus 4% among adults, *P* < 0.05). Children’s specimens were more often stored in the local laboratory’s refrigerator than adults’ specimens (78% versus 58%, *P* < 0.05). For specimens stored in the refrigerator, the median storage time in the local laboratory was longer than for specimens stored at ambient temperature (*P* < 0.001, Table 1).

**TABLE 1:** Characteristics and storage time and conditions of stool samples included by age group of participants^*a*^

Characteristic of stool	All participants	Children	Adults
**Per participant analysis**	***n* (%)**	***n* (%)**	***n* (%)**
No. of participants	47	11	36
No. of samples collected per participant, median (range)	3 (1–4)	3 (1–4)	3 (1–3)
Median interval between collection of first stool sample and start of TB treatment, days (IQR)^*b*^	0 (−1–1)	−1 (−2–4)	0 (–1–1)
Median interval between collection of the last stool sample and start of TB treatment, days (IQR)^*b*^	2 (1–3)	2 (0–4)	2 (1–3)
**Per stool analysis**			
Total no. of stool specimens collected	132	27	105
Consistency			
Formed (solid)	26 (19.7)	8 (29.6)	18 (17.1)
Semiformed (soft)	105 (79.6)	19 (70.4)	86 (81.9)
Taking shape of container (liquid)	1 (0.8)	0 (0)	1 (1.0)
Wt (g) of specimen, median (IQR)	29.5 (22.9–37.7)	25.6 (17.4–42.4)	30.7 (24.9–37.5)
Where was the specimen collected?^*c*^			
On the spot by a nurse	10 (7.6)	2 (7.4)	8 (7.6)
On the spot by participant/caregiver	111 (84.1)	18 (66.7)	93 (88.6)
At home	11 (8.3)	7 (25.9)	4 (3.8)
Median time from collection at home until receipt in local clinic, minutes	28.4 (19.7–240.3)	19.7 (19.7–135.4)	195.5 (83–264.3)
How was the specimen stored in the clinic?			
At ambient temp	50 (37.9)	6 (22.2)	44 (41.9)
In the refrigerator	82 (62.1)	21 (77.8)	61 (58.1)
Mean storage temp, °C (SD)^*c*^	4.4 (0.9)	5.0 (1.1)	4.1 (0.6)
Median duration of storage until transportation to NTRL (hours)	3.5 (1.1–23.9)	3.1 (1.2–22.9)	3.7 (1.1–24.0)
If stored at ambient temp	1.1 (0.4–3.7)^*b*^	1.7 (0–3.1)	1.1 (0.4–4.1)
If stored in refrigerator	10.1 (2.1–26.0)^*b*^	3.9 (2.0–25.2)	19.0 (2.3–26.5)
Median temp of specimen during transport to NTRL, °C (SD)^*c*^	5 (5–6)	5 (4–6)	6 (5–6)
Median delay between stool collection and registration for experiments (hours)	4.8 (2.9–25.0)	4.6 (1.8–23.5)	5.2 (3.0–25.0)

a IQR, interquartile range; No., number; NTRL, National Tuberculosis Reference Laboratory; SD, standard deviation; temp, temperature; Wt, weight.

b *P* < 0.001, significantly different.

c *P* < 0.05 for difference between age groups.

**FIG 2:**
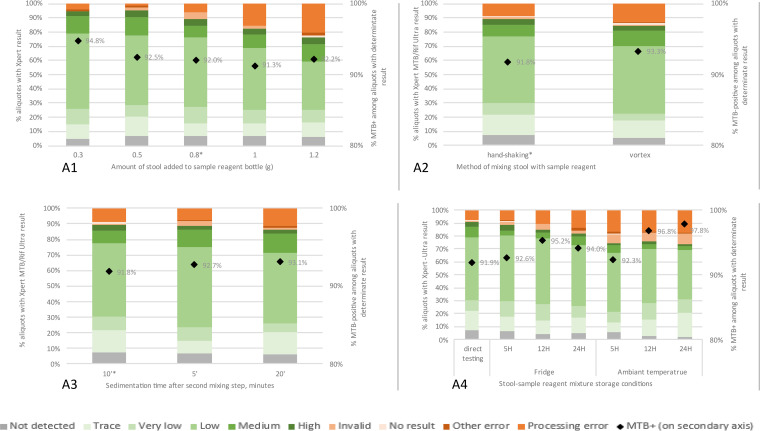
Xpert-Ultra results after modifications to the standard SOS stool processing protocol. Each chart represents one experiment, in which one step in the standard SOS protocol was varied. In experiment A4, the standard protocol was interrupted after the first hand-shaking step, after which the sample was stored for the indicated time and at the indicated temperature. For experiments A1 to A3, the results after processing according to the standard SOS stool protocol are indicated with an asterisk (*). Xpert-Ultra results after various stool transport conditions before starting the SOS stool processing for Xpert mimicking sample transport conditions.

A total of 84 stools from 43 participants were included in experiment A1 (Table 2). The proportion of stool processing errors increased significantly from 3.7% to 20.2% at 0.3 g and 1.2 g stool, respectively, while MTB positivity rate remained the same (Fig. 2, Table 3). These processing error rates strongly increased when adding more than 0.8 g stool (Table 3 and Fig. S2 in the supplemental material).

**TABLE 2:** Overview of the experiments and number of stools used for stool processing (robustness) and stool specimen transport of the SOS stool processing method^*a*^

Experiment or procedure step		No. of participants	No. of stools	No. of aliquots	Standard protocol	Added experiments/modified protocol
**A. Stool processing (robustness)**						
	A1. Stool volume	43	84	420^*b*^		0.3 g (replacing 1.5 g)
						0.5 g
					0.8 g	
						1.0 g
						1.2 g
	A2. Mixing stool/SR by handshake or vortex	44	123	246	Handshake 30 seconds (2×)	Vortex 30 seconds (2×)
	A3. Sedimentation time	44	123	364^*c*^		5 minutes
					10 minutes	
						20 minutes
	A4. Storage conditions stool/SR mixture	43	122	854		2–8°C; 5, 12, and 24 h
					2–8°C; 4 h	RT; 5, 12, and 24 h
						37°C; 5, 12, and 24 h
**B. Stool transport**						
	Stool transport from collection to testing				2–8°C; 120 h	2–8°C; 48, 72, and 240 h
		42	83	1,079	RT; 48 h	RT; 72, 120, and 240 h
						37°C; 48, 72, 120, and 240 h

a No., number; RT, room (or ambient) temperature; SR, sample reagent.

b Three aliquots were subjected to an experiment using 1.5 g of stool, but the team had experienced that it was better to use less instead of more than 1 g of stool; this amount was replaced by adding 0.3 g.

c Five aliquots were subjected to an experiment in which sedimentation time was 15 min overall, without a second shaking step. Then, the 15-min experiment was revised and replaced with sedimentation for 20 min, after the second shaking step.

**TABLE 3:** Association of stool specimen transport conditions and modified stool processing on the rate of errors associated with filter blockage and/or fluid transfer and on the MTB-positivity rate^*a*^

Experiment	No. of aliquots			Association with errors related to filter blockage/fluid transfer^*b*^		Association with MTB-positivity^*c*^	
	Processed and tested	With valid result	With MTB detected^*c*^	Univariable or (95% CI)	Multilevel or (95% CI)§	Univariable or (95% CI)	Multilevel or (95% CI)§
A1. Stool vol				18.7 (4.93–70.7)¶		0.7 (0.19–2.2)¶	0.6 (0.08–4.9)¶, ¶¶
0.3 g	81£	77	73	0.61 (0.14–2.63)		1.59 (0.43–5.87)	
0.5 g	84	80	74	0.19 (0.02–1.67)		1.07 (0.33–3.48)	
0.8 g	84	75	69	1 (REF)		1 (REF)	
1.0 g	84	69	63	2.89 (0.98–8.52)		0.91 (0.28–2.98)	
1.2 g	84	64	59	4.01 (1.4–11.44)		1.03 (0.3–3.53)	
A2. Mixing stool and SR							
Handshaking	123	110	101	1 (REF)	1 (REF)¶¶	1 (REF)	
Vortexing	123	104	97	1.52 (0.68–3.43)	1.86 (0.68–5.12)	1.23 (0.44–3.45)	
A3. Sedimentation time							
5 min	123	110	101	1 (REF)	1 (REF)¶¶	1 (REF)	
10 min	123	109	101	0.8 (0.32–2.01)	0.73 (0.24–2.2)	1.13 (0.42–3.03)	
20 min	118∞	102	95	1.26 (0.54–2.94)	1.51 (0.54–4.23)	1.21 (0.43–3.38)	
A4. Storage of stool/SR mixture‡							
Direct testing (no storage)	122	111	102	1 (REF)	1 (REF)¶¶	1 (REF)	
Fridge							
5 h	122	108	100	1 (0.38–2.61)	1 (0.35–2.89)	1.1 (0.41–2.97)	
12 h	122	104	99	1.5 (0.62–3.65)	1.66 (0.61–4.48)	1.75 (0.57–5.4)	
24 h	122	100	94	2.03 (0.87–4.76)	2.46 (0.94 - 6.42)	1.38 (0.47–4.03)	
Room temp							
5 h	122	91	84	2.46 (1.07–5.65)	3.17 (1.24 - 8.13)	1.06 (0.38–2.96)	
12 h	122	93	90	2.46 (1.07–5.65)	3.17 (1.24 - 8.13)	2.65 (0.7–10.08)	
24 h	122	90	88	2.61 (1.14–5.96)	3.43 (1.35 - 8.76)	3.88 (0.82–18.45)	
B. Stool specimen transport conditions†							
No simulated transport	83	78	72	1 (REF)	1 (REF)	1 (REF)	
Fridge							
48 h	83	75	68	1.27 (0.33–4.89)	1.32 (0.31–5.67)	0.81 (0.26–2.53)	
72 h	83	73	66	2.11 (0.61–7.28)	2.44 (0.63–9.43)	0.79 (0.25–2.46)	
120 h	83	74	69	1.54 (0.42–5.66)	1.66 (0.4–6.83)	1.15 (0.34–3.94)	
240 h	83	73	69	1.54 (0.42–5.66)	1.66 (0.4–6.83)	1.44 (0.39–5.31)	
Room temp							
48 h	83	63	60	5.45 (1.76–16.9)	8.45 (2.42–29.5)	1.67 (0.4–6.95)	
72 h	83	71	68	2.7 (0.81–8.99)	3.34 (0.9–12.4)	1.89 (0.45–7.85)	
120 h	83	68	67	4 (1.26–12.7)	5.57 (1.56–19.9)	5.58 (0.65–47.6)	
240 h	83	64	59	4.71 (1.5–14.7)	6.92 (1.96–24.4)	0.98 (0.29–3.38)	
Incubator							
48 h	83	65	63	4.35 (1.38–13.7)	6.22 (1.76–22.1)	2.63 (0.51–13.47)	
72 h	83	59	57	5.85 (1.89–18)	9.3 (2.67–32.3)	2.38 (0.46–12.21)	
120 h	83	62	61	4.35 (1.38–13.7)	6.22 (1.76–22.1)	5.08 (0.6–43.39)	
240 h	83	60	58	6.25 (2.03–19.2)	10.2 (2.94–35.3)	2.42 (0.47–12.42)	

a Multilevel analyses are displayed if these models performed better than the crude models. †, conditions after receipt at NTRL, so excluding the conditions occurring between stool collection and receipt at the NTRL; §, multilevel logistic regression analysis, including two levels as results were considered to be correlated within participants and stools, unless otherwise indicated; ¶, shows the trend per step of 0.2 g. Multilevel logistic regression analysis, including two levels as results were considered to be correlated within stools within participants; ¶¶, multilevel logistic regression analysis, including one level as results showed to be correlated within participants, but not within stools of the same participant; £, 3 samples were used for an early experiment with 1.5 g of stool; ∞, 5 aliquots were used for an early “no sedimentation” experiment.

b These are errors 2008, 5006, 5007, and 5017, which may be related to stool processing, as these may be caused by debris clogging the cartridge filter (2008) or disturbing the fluid transfer within the cartridge (5006, 5007, and 5017).

c MTB positivity defined as trace call or higher.

We included 123 stool samples from 43 participants in experiment A2. There was no significant difference in the rate of stool processing errors or MTB positivity between using the vortex and handshaking (Fig. 2, Table 3).

The same 123 stool specimens as in experiment A2 were used for experiment A3. Fig. 2 and Table 3 show that variations in sedimentation time between 5, 10, and 20 min did not affect the rate of errors related to stool processing or MTB-positive Xpert-Ultra results.

For experiment A4, we used a total of 122 stools from 43 participants, which were also included in experiment A2. As with the stool storage experiment, the temperature at which the mixture was stored had a greater impact on the rate of processing errors than the time (*P* = 0.02 for storage in the fridge versus for storage at room temperature (RT); *P* = 0.08 and *P* = 0.03 for storage up to 12 and 24 h, respectively, versus no storage) (Table 3, Table S2). There was no effect of storage on MTB positivity rate. However, the rate of invalid results increased when stored for >5 h and/or at RT (Fig. 2). Also, after 24 h of storage, the rate of MTB-positive RIF indeterminate results was higher than for shorter storage times (5.0% versus 1.3%, *P* = 0.01) (Table S3).

**FIG 3:**
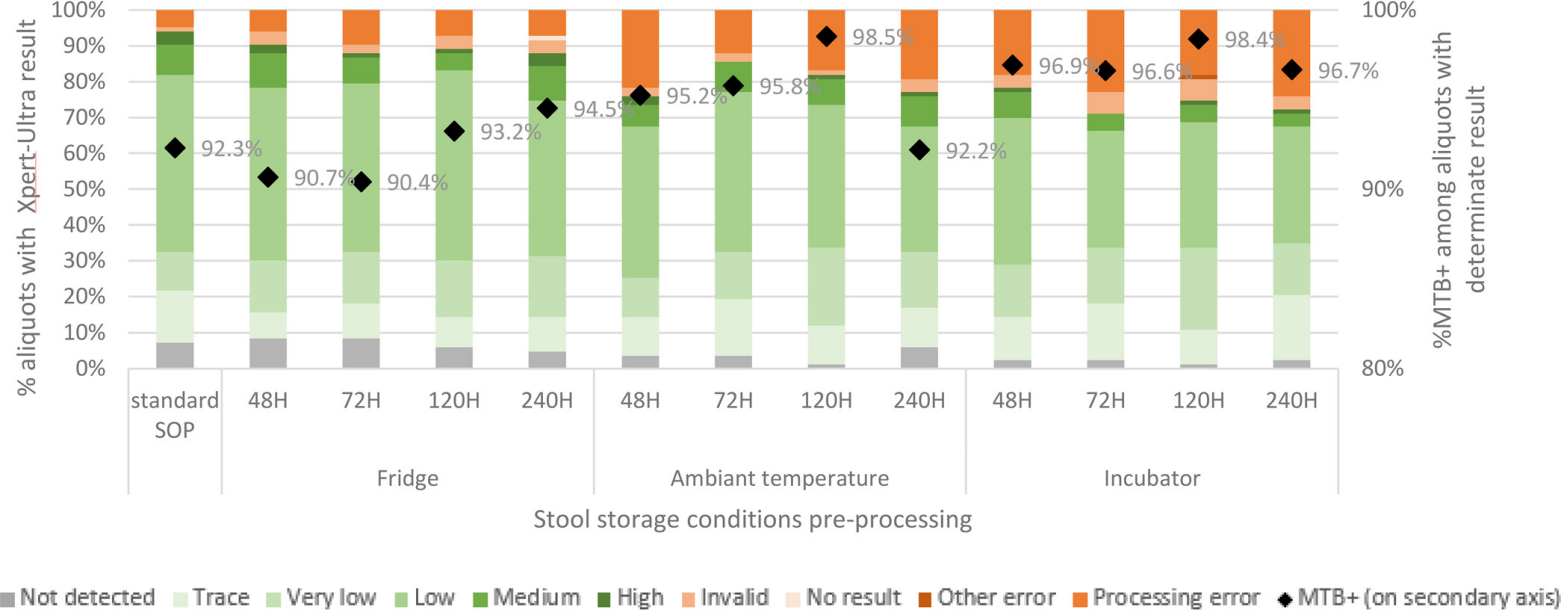
Xpert-Ultra results after various stool transport conditions before starting the SOS stool processing for Xpert mimicking sample transport conditions.

Eighty-four stool samples from 42 participants were available to evaluate the effect of stool transport conditions (experiment B). Fig. 3 shows the proportion of Xpert-Ultra results per stool specimen transport condition. Temperature had a bigger impact on the Xpert-Ultra processing error rate than time (Fig. 3, Table 3). Stratifying the analysis for time showed significantly increased risks for processing errors in aliquots stored at RT and in the incubator, while stratifying for temperature showed no effect of storage time, even if stored up to 240 h (Table S1).

We found no clear relationship between any of the Ct values and experimental conditions. Also, except for experiment A4, the experimental conditions did not affect the rate of invalid results (Fig. 2 and 3).

## DISCUSSION

In this study, we conducted Xpert-Ultra tests to evaluate the robustness of the SOS stool processing method and simulated stool transport conditions mimicking routine conditions in resource limited settings using stool specimens of TB patients. Most variations in stool processing did not significantly affect the occurrence of processing errors, nor of invalid, MTB-positive or RIF indeterminate results. Stool processing error rates increased when stool amount exceeded 0.8 g. Mixing stool with sample reagent (SR) by handshaking or vortexing, and mixture sedimentation time (5, 10, and 20 min) did not significantly affect the Xpert-Ultra test results. Exposing stool to SR for more than 5 h and/or at RT increased the rate of invalid results, while exposure for more than 24 h increased the possibility of RIF indeterminate results. Regarding stool transport conditions, keeping stool at RT or above led to a higher rate of processing errors, while storage time had no significant impact.

In this study, instead of nondeterminate results, we focused on errors potentially related to the processing of stool. Stool contains more debris than sputum, which increases risk of microfluid filter blockage in the cartridge and increases the pressure within-cartridge above the threshold (120 lb/in^2^ for Xpert, 100 lb/in^2^ for Xpert-Ultra). Indeed, errors related to this aspect (2008, 5006, 5007) were most reported when the SOS stool method was applied (6, 9), including in this study. The results showed that the rate of invalid results was not associated with stool processing conditions, except for longer exposure of stool to SR. Invalid results (likely related to PCR inhibition) are more logically related to stool characteristics than to the processing conditions.

Adding more than 0.8 g of stool to SR led to a significant increase in stool processing errors, while the MTB positivity rate remained unaffected by the stool quantity. This is different from our findings during the validation phase of the SOS stool processing method (6), which suggested that a higher stool volume would increase the yield of MTB, while error rates also increased. Based on this, we recommended adding the maximum quantity of stool with an acceptable rate of nondeterminate results (0.8 g). The current study disproves this assumption: there was no association between semiquantitative Xpert-Ultra results and stool quantity. Therefore, we conclude that the exact amount of stool added is not very precise, which justifies the visual picking of stool instead of weighing, as recommended in the standard SOS stool processing method protocol (6). However, to prevent a high rate of unsuccessful results, the stool amount should not exceed 0.8 g. Based on these results, we modified the SOS stool processing protocol to use a wider range of stool volume (0.3 to 0.8 g).

Mixing stool/SR by handshaking or vortexing yielded comparable Xpert-Ultra results, although a nonsignificantly higher rate of processing errors was found using vortexing compared to handshaking (13% versus 9%). Higher forces of the vortex on the stool/SR mixture may increase the breakdown of organic material into fine debris. These findings emphasize that the current recommendation of handshaking suffices, and no additional equipment is required to enhance the shaking force.

We expected a shorter sedimentation time would lead to more debris in the supernatant, increasing the risk of stool processing errors. However, neither shorter (5 min) nor longer (20 min) sedimentation than currently recommended (10 min) had a significant effect on Xpert-Ultra results. This finding supports our recommendation to allow for longer sedimentation for samples with partially settled debris (11), and resediment for 5 min if the laboratory personnel was unable to aspirate 2 mL of supernatant in one go.

Storing the stool/SR mixture for up to 24 h or at RT increased the risk of RIF indeterminate results, which is consistent with another study (12). This could be because the high concentration of NaOH in the SR degrades DNA fragments, making it more difficult to detect the *rpoB* probes. Also, the risk of processing errors and invalid Xpert-Ultra results increased. Possibly, longer exposure of stool to SR leads to degradation of organic materials into fine debris and releases of substances from debris into the supernatant, which may inhibit the PCR. Further investigation will be needed to better understand this mechanism.

Stool transport at higher temperatures significantly increased the rate of processing errors in the Xpert-Ultra test. This could be due to temperature-dependent degradation of organic materials and ongoing transformation of stool microbiomes (growth-death). Sedimentation of this fine debris may be more challenging and increase the risk of microfluid filter blockage in the cartridge, leading to higher processing error rate. MTB positivity slightly increased with higher stool transport temperatures, although this was not statistically significant. This suggests that the degradation process may facilitate the release of MTB from organic materials. Surprisingly, stool storage time did not importantly impact the stool processing error rates. This may suggest that the temperature-dependent release of fine debris occurs within a few hours after which an equilibrium is reached with a concentration of fine debris that causes pressure above the threshold level within-cartridge.

To our knowledge, this is the first study that determined the robustness of the SOS stool processing method and evaluated stool transport conditions. We included nearly 3,000 Xpert-Ultra test results from multiple specimens/aliquots of the same person, which enabled us to control potential confounders more effectively. There are some limitations to this study. First, the study does not provide guidance for liquid stool specimens and the generalizability of our results to children may be limited due to a paucity of data, although current evidence does not suggest that the SOS stool method would behave differently on children compared to adult stool (9). Second, we did not monitor the storage conditions of stool specimens that were collected at the homes of participants. However, almost all stool specimens (92%) were collected in the clinic. Third, we did not assess the effect on Xpert-Ultra results of storage of stools in the freezer. Last, initial analysis of the Ct values did not indicate any clear associations between these and experimental conditions. In-depth analysis may be performed to better understand these data. It should be noted that global routine implementation of Xpert stool testing may lead to further deviations from the standard SOS stool processing method than tested in our study. However, ongoing implementation has not invalidated the robustness of the method (9).

In conclusion, minor deviations from the standard SOS stool protocol generally did not impact the Xpert-Ultra results. The only adjustment to be made based on this study, is to use a wider range of the amount of stool (0.3 to 0.8 g) than advised previously (0.8 g), as this leads to a lower rate of stool processing errors while not affecting MTB positivity rate. Further, it is advisable to keep the stool as much as possible in a cold chain after collection and during transport to avoid an increase in processing errors. However, it should be noted that reliable Xpert-Ultra results were obtained even after 240 h of storage at 37^°^C. We conclude that the SOS stool processing method for stool Xpert testing is ready for global scale-up.

## MATERIALS AND METHODS

### Study design and setting.

This cross-sectional study was conducted in Addis Ababa, Ethiopia, from December 2019 to March 2022. A detailed description of the study protocol, including experiments, was published elsewhere (10). The participants were enrolled from 23 health facilities in Addis Ababa (7 hospitals and 16 health centers), selected for their relatively high TB notification rate and experience with research and availability of a GeneXpert instrument at the facility level. Children were included among participants in the Alternatives to Sputum Testing for Tuberculosis in Indonesia and Ethiopia (ASTTIE) study. To reach the desired sample size, adult participants who tested MTB-positive on sputum were enrolled from the same facilities. This laboratory study was conducted at the National TB Reference Laboratory (NTRL) of Ethiopian Public Health Institute (EPHI) and was part of a project called Painless Optimized Diagnosis of Tuberculosis in Ethiopian Children (PODTEC).

### Sample size and sampling.

To assess the impact of modifications to the SOS stool standard protocol and of different transport conditions, we estimated that 50 participants would be needed for this study, using McNemar’s test for paired proportions with a study power of 80% and an alpha-level of 95% (10). Three stool specimens were collected on consecutive days from bacteriologically confirmed MTB positive children (age ≤ 10 years) (sputum/NGA and/or stool Xpert-Ultra MTB-positive) and adults (sputum Xpert MTB-positive) who had not received anti-TB treatment for more than 5 days. The participants or caregivers were provided with stool containers at the time of diagnosis to allow the collection of at least 30 g of stool. If collection was done at home, they were instructed to protect the stool specimens against daylight and heat and bring them to the health facility within 24 h of collection. At the health facilities, the collected stool samples were stored refrigerated (2 to 8°C) or at ambient temperature until transportation to EPHI. A stool submission form was completed at the stool collection site, and a unique participant identification code was printed and pasted on the stool submission forms and stool containers. The stool specimens were triple packed and transported in a cold chain to EPHI on the first transportation opportunity, and the temperature in the transport box was recorded upon arrival at the NTRL. Also, the date and time of arrival, volume, and consistency of the stool specimen were recorded on the stool submission form.

### Laboratory procedures.

After registration, the stool specimen was split into multiple aliquots. Formed and semiformed stool specimens were split into aliquots of 0.8 g; liquid stools were split into aliquots of 2 ml. Stool aliquots were then assigned to the specific experiments using a flow diagram (10), providing for prioritization of experiments in case not enough stool was provided to perform all the experiments. All experiments were performed by two laboratory experts who were involved in the development of the SOS stool processing method and had extensive testing experience.

### Experiments.

The first set of experiments (A) aimed to investigate the robustness of the SOS stool method, whereby specific steps of the standard SOS procedure were modified, and the second set (B) was designed to investigate optimal stool specimen transport conditions (Table 2 and Fig. S1). For the robustness experiments (A) modifications to the stool volume (A1), shaking method (A2), sedimentation time (A3), and contact time of stool with the SR at different temperatures (A4) were investigated.

The standard SOS stool processing method involves adding 0.8 g or 2 mL of stool to the sample reagent (SR) bottle, handshaking for 30 s, incubation for 10 min, followed by handshaking again for 30 s and incubation for another 10 min to allow debris to settle (this 10-min step is referred to as sedimentation time or step), and transfer of 2 mL of the supernatant to the Xpert-Ultra cartridge to run the test using the GeneXpert instrument (6).

### (i) A1. Stool volume.

To investigate the optimum stool volume, five aliquots of different amounts (0.3, 0.5, 0.8 [standard procedure], 1.0, and 1.2 g of stool) per stool were each added to the SR bottle and processed according to the standard SOS stool method.

### (ii) A2. Handshaking versus vortex.

To compare shaking by hand to using a vortex (mimicking more forceful mixing) to mix the stool with the SR, two aliquots per stool were each added to separate SR bottles. The mixture was then processed following the standard SOS stool method, with the difference that one SR bottle was hand-shaken for 2 × 30 s (standard procedure), while the other bottle was mixed using a vortex for 2 × 30 s.

### (iii) A3. Sedimentation time.

To investigate the optimum and maximum sedimentation times, three aliquots per stool specimen were each added to separate SR bottles. The mixture was processed following the standard SOS stool method, with the difference that one bottle was left for sedimentation of 5 min, one for 10 min (standard procedure), and one for 20 min.

### (iv) A4. Stool/SR mixture storage conditions.

To investigate the optimum and maximum stool/SR mixture storage conditions, six stool aliquots per stool were added into separate SR bottles, hand-shaken for 30 s and then stored in the refrigerator (2 to 8°C; 3 bottles) or at temperature (RT) (20 to 25°C; 3 bottles). After 5, 12, and 24 h, for each storage temperature, one bottle was taken and processed further using the standard SOS stool method. The results were compared to those obtained after applying the standard procedure in experiment A2.

### (v) B. Stool specimen transport conditions.

To investigate the optimal transport conditions, 12 aliquots per participant were stored at three different temperatures: four in the refrigerator (2 to 8°C), mimicking cold-chain transport; four at RT (20 to 25°C); and four in the incubator (37^°^C), mimicking transport in hot climates. After 48, 72, 120, or 240 h, for each storage temperature, one aliquot was taken and processed using the standard SOS stool method.

### Data management and statistical analysis.

At enrollment, from each individual, demographic and treatment information was collected (age, sex, date of TB diagnosis, date of starting TB treatment) on the participation form. Furthermore, specific data on stool collection (place, date, and time), storage until transport to EPHI (place, temperature, and time), transport conditions (temperature and time), and stool characteristics (appearance, weight) were recorded on standard stool submission forms. Experimental conditions and Xpert-Ultra results (semiquantitative results as well as Ct values for all probes) were captured on laboratory forms. All paper forms were created as prestructured EpiData files (EpiData version 3.1; www.epidata.dk) and the collected data were entered into it. After validation, the data set was transferred to Stata 15.0/SE (StataCorp LCC, TX, USA) for analysis.

Descriptive analysis was used to describe the stool characteristics and experimental outcomes. For each experiment, we compared the MTB positivity rate (i.e., number of aliquots in which MTB was detected, any semiquantitative result, including MTB trace, Very Low, Low, Medium, and High results divided by the total number of aliquots with valid Xpert-Ultra test results), the rate of invalid results, and the rate of errors possibly related to stool processing (i.e., number of aliquots returning error codes 2008 (potentially caused by debris clogging the cartridge filter), 5006, 5007, and 5017 (potentially caused by disturbing the fluid transfer within the cartridge) divided by the total number of aliquots tested) obtained for the standard SOS procedure against the modified procedures. Other errors and “no result” were considered unrelated to stool processing and thus were not considered useful outcomes. Unsuccessful Xpert-Ultra results (invalids/error/no result) were not repeated purposively as we considered them to be test outcomes. We also assessed the potential association between Ct values and experimental conditions.

Univariable and multivariable logistic regression analyses were conducted to assess the impact of factors related to the experiments (e.g., storage time and temperature), and stool (e.g., consistency, time between collection and experiments) on the MTB-positive, processing error, or invalid result (outcome). We applied multilevel analysis to account for the fact that stool specimens were clustered within participants, and aliquots within stools.

The Xpert-Ultra stool result from each aliquot was compared with the Xpert-Ultra result following the standard protocol. Trends in MTB-positivity, processing errors and invalid rates over, e.g., increasing storage time or temperature, or increasing amounts of stool added, were analyzed using the Wilcoxon-like test for trend across ordered groups using nptrend in Stata (13). In this study, we defined “robustness” as minor deviations from the standard SOS stool processing protocol (experiments A1, A2, A3, and A4) that did not significantly affect the Xpert-Ultra test outcomes.

### Ethical considerations.

The study protocol was approved by the Ethical Review Board of the Ethiopian Public Health Institute (EPHI-IRB-234-2020). Eligible persons were informed about the study and received an information sheet with study details. Written informed consent, parental consent, or assent (depending on the age of participant) was obtained if they agreed to participate in the study. Participants’ information was kept confidential. The data set for analysis did not contain any personal identifying information and used unique personal identification codes instead.

## References

[1] WHO. 2022. WHO operational handbook on tuberculosis. Module 5: management of tuberculosis in children and adolescents. World Health Organization, Geneva, Switzerland. 35404556

[2] Basile FW, Nabeta P, Ruhwald M, Song R. 2022. Pediatric tuberculosis diagnostics: present and future. J Pediatric Infect Dis Soc 11:S85–S93. doi:10.1093/jpids/piac082. 36314546 PMC9620430

[3] WHO. 2021. WHO operational handbook on tuberculosis. Module 3: diagnosis - rapid diagnostics for tuberculosis detection, 2021 update. World Health Organization, Geneva, Switzerland.

[4] WHO. 2022. Practical manual of processing stool samples for diagnosis of childhood TB. World Health Organization, Geneva, Switzerland.

[5] Mafirakureva N, Klinkenberg E, Spruijt I, Levy J, Shaweno D, De Haas P, Kaswandani N, Bedru A, Triasih R, Gebremichael M, Dodd PJ, Tiemersma EW. 2022. Xpert Ultra stool testing to diagnose tuberculosis in children in Ethiopia and Indonesia: a model-based cost-effectiveness analysis. BMJ Open 12:e058388. doi:10.1136/bmjopen-2021-058388. PMC9252203 35777870

[6] de Haas P, Yenew B, Mengesha E, Slyzkyi A, Gashu Z, Lounnas M, Tesfaye E, Bedru A, Tiemersma E, Kremer K, Amare M, Diriba G, Zerihun B, Gudina T, Tegegn B, Bonnet M, Negeri C, Klinkenberg E. 2021. The Simple One-Step (SOS) stool processing method for use with the Xpert MTB/RIF assay for a child-friendly diagnosis of tuberculosis closer to the point-of-care. J Clin Microbiol 59:e00406-21. doi:10.1128/JCM.00406-21. 34076469 PMC8373220

[7] Jasumback CL, Dlamini Q, Kahari J, Maphalala G, Dlamini MG, Dube GS, DiNardo A, Kirchner HL, Mandalakas A, Kay AW. 2021. Laboratory comparison of stool processing methods for Xpert Ultra. Public Health Action 11:55–57. doi:10.5588/pha.20.0079. 34159062 PMC8202631

[8] Dubale M, Tadesse M, Berhane M, Mekonnen M, Abebe G. 2022. Stool-based Xpert MTB/RIF assay for the diagnosis of pulmonary tuberculosis in children at a teaching and referral hospital in Southwest Ethiopia. PLoS One 17:e0267661. doi:10.1371/journal.pone.0267661. 35511771 PMC9070927

[9] de Haas P, Nhung NV, Hng NT, Hoà NB, Loan NB, Thanh NTK, Gebhard A, Slyzkyi A, Tue PQH, Ng NV, Hang PT, Ngoc KTT, Tiemersma EW. 2023. Introduction of the Simple One-Step stool Xpert Ultra method to detect TB in children and adults. Int J Tuber Lung Dis 27:19–27. doi:10.5588/ijtld.22.0161. 36853124

[10] de Haas P, Yenew B, Diriba G, Amare M, Slyzkyi A, Demissie Y, Sherefdin B, Bedru A, Mengesha E, Dememew ZG, Kebede A, Getahun M, Tiemersma E, Jerene D. 2022. The Simple One-step stool processing method for detection of Pulmonary tuberculosis: a study protocol to assess the robustness, stool storage conditions and sampling strategy for global implementation and scale-up. PLoS One 17:e0264103. doi:10.1371/journal.pone.0264103. 36194578 PMC9531811

[11] KNCV Tuberculosis Foundation. 2021. SOS Stoolbox: simple one step (SOS) stool processing method and Xpert MTB/RIF (Ultra) testing for the detection of *Mycobacterium tuberculos* is complex and rifampicin resistance. Standard operating procedure (SOP). The Hague: KNCV Tuberculosis Foundation (https://www.kncvtbc.org/uploaded/2021/03/Stoolbox-SOP1.pdf).

[12] Banada PP, Sivasubramani SK, Blakemore R, Boehme C, Perkins MD, Fennelly K, Alland D. 2010. Containment of bioaerosol infection risk by the Xpert MTB/RIF assay and its applicability to point-of-care settings. J Clin Microbiol 48:3551–3557. doi:10.1128/JCM.01053-10. 20720033 PMC2953088

[13] Cuzick J. 1985. A Wilcoxon-type test for trend. Stat Med 4:87–90. doi:10.1002/sim.4780040112. 3992076

