# Supplementary material for: Optimization of the Simple One-Step Stool Processing Method to Diagnose Tuberculosis: Evaluation of Robustness and Stool Transport Conditions for Global Implementation
Source: Microbiol Spectr. 2023 Jun 26;11(4):e01171-23. doi: 10.1128/spectrum.01171-23 (PMC10434014; doi:10.1128/spectrum.01171-23)
Supplement: Supplemental file 1 — Table S1. Download spectrum.01171-23-s0001.docx, DOCX file, 0.02 MB [file spectrum.01171-23-s0001.docx]

**Supplement Table 1.** Stratified analysis of stool transport conditions experiment (B). Odds ratios (ORs) and 95% confidence intervals (95%CI) are shown. Bold figures represent statistically significant

|  |  | **OR (95%CI) for the association with** | | |
| --- | --- | --- | --- | --- |
|  |  | **Processing error** | **MTB+** | **Invalid result** |
| **Stratified by storage time** | |  |  |  |
| 48 H | Fridge | 1 (REF) | 1 (REF) | 1 (REF) |
|  | RT | **4.3 (1.5 - 12.3)** | 2.1 (0.5 - 8.3) | 0.7 (0.1 - 4) |
|  | Incubator | **3.4 (1.2 - 10)** | 3.2 (0.6 - 16.2) | 1.0 (0.2 - 5.1) |
| 72 H | Fridge | 1 (REF) | 1 (REF) | 1 (REF) |
|  | RT | 1.3 (0.5 - 3.4) | 2.4 (0.6 - 9.7) | 1.0 (0.1 - 7.3) |
|  | Incubator | **2.8 (1.1 - 6.8)** | 3 (0.6 - 15.1) | 2.6 (0.5 - 13.8) |
| 120 H | Fridge | 1 (REF) | 1 (REF) | 1 (REF) |
|  | RT | 2.6 (0.9 - 7.1) | 4.9 (0.6 - 42.7) | 0.3 (0 - 3.2) |
|  | Incubator | **2.8 (1.0 - 7.7)** | 4.4 (0.5 - 38.9) | 1.7 (0.4 - 7.4) |
| 240 H | Fridge | 1 (REF) | 1 (REF) | 1 (REF) |
|  | RT | **3.1 (1.1 - 8.3)** | 0.7 (0.2 - 2.7) | 1.0 (0.2 - 5.1) |
|  | Incubator | **4.1 (1.5 - 10.8)** | 1.7 (0.3 - 9.5) | 1.0 (0.2 - 5.1) |
| **Stratified by storage temperature** | |  |  |  |
| Fridge | 48 H | 1 (REF) | 1 (REF) | 1 (REF) |
|  | 72 H | 1.7 (0.5 - 5.3) | 1.0 (0.3 - 2.9) | 0.7 (0.1 – 4.0) |
|  | 120 H | 1.2 (0.4 - 4.1) | 1.4 (0.4 - 4.7) | 1.0 (0.2 - 5.1) |
|  | 240 H | 1.2 (0.4 - 4.1) | 1.8 (0.5 - 6.3) | 1.0 (0.2 - 5.1) |
| RT | 48 H | 1 (REF) | 1 (REF) | 1 (REF) |
|  | 72 H | 0.5 (0.2 - 1.1) | 1.1 (0.2 - 5.8) | 1.0 (0.1 - 7.3) |
|  | 120 H | 0.7 (0.3 - 1.6) | 3.3 (0.3 - 33.1) | 0.5 (0.0 - 5.6) |
|  | 240 H | 0.9 (0.4 - 1.8) | 0.6 (0.1 - 2.6) | 1.5 (0.2 - 9.3) |
| Incubator | 48 H | 1 (REF) | 1 (REF) | 1 (REF) |
|  | 72 H | 1.3 (0.6 - 2.9) | 0.9 (0.1 - 6.6) | 1.7 (0.4 - 7.4) |
|  | 120 H | 1.0 (0.5 - 2.2) | 1.9 (0.2 - 21.9) | 1.7 (0.4 - 7.4) |
|  | 240 H | 1.4 (0.7 - 3.1) | 0.9 (0.1 - 6.7) | 1.0 (0.2 - 5.1) |
