# Supplementary material for: Optimization of the Simple One-Step Stool Processing Method to Diagnose Tuberculosis: Evaluation of Robustness and Stool Transport Conditions for Global Implementation
Source: Microbiol Spectr. 2023 Jun 26;11(4):e01171-23. doi: 10.1128/spectrum.01171-23 (PMC10434014; doi:10.1128/spectrum.01171-23)
Supplement: Supplemental file 2 — Table S2. Download spectrum.01171-23-s0002.docx, DOCX file, 0.01 MB [file spectrum.01171-23-s0002.docx]

**Supplement Table 2.** Stratified analysis of stool/SR mixture storage conditions experiment (A4).

|  |  | **OR (95% CI) for the association with** | | |
| --- | --- | --- | --- | --- |
|  | | **Processing error** | **MTB+** | **Invalid result** |
| **Stratified by storage time** | |  |  |  |
| 5 H | Fridge | 1 (REF) | 1 (REF) | 1 (REF) |
|  | RT | **2.5 (1.1 - 5.7)** | 1 (0.3 - 2.8) | 2.8 (0.7 - 10.8) |
| 12 H | Fridge | 1 (REF) | 1 (REF) | 1 (REF) |
|  | RT | 1.6 (0.8 - 3.5) | 1.5 (0.4 - 6.5) | 1.6 (0.5 - 5.2) |
| 24 H | Fridge | 1 (REF) | 1 (REF) | 1 (REF) |
|  | RT | 1.3 (0.6 - 2.6) | 2.8 (0.6 - 14.3) | 3.5 (0.95 - 13.2) |
| **Stratified by storage temperature** | | |  |  |
| Fridge | 5 H | 1 (REF) | 1 (REF) | 1 (REF) |
|  | 12 H | 1.5 (0.6 - 3.6) | 1.6 (0.5 - 5) | 1.7 (0.4 - 7.3) |
|  | 24 H | 2 (0.9 - 4.8) | 1.3 (0.4 - 3.7) | 1 (0.2 - 5.1) |
| RT | 5 H | 1 (REF) | 1 (REF) | 1 (REF) |
|  | 12 H | 1 (0.5 - 2) | 2.5 (0.6 - 10) | 1 (0.4 - 2.8) |
|  | 24 H | 1.1 (0.5 - 2.1) | 3.7 (0.7 - 18.2) | 1.3 (0.5 - 3.3) |
