# Supplementary material for: Optimization of the Simple One-Step Stool Processing Method to Diagnose Tuberculosis: Evaluation of Robustness and Stool Transport Conditions for Global Implementation
Source: Microbiol Spectr. 2023 Jun 26;11(4):e01171-23. doi: 10.1128/spectrum.01171-23 (PMC10434014; doi:10.1128/spectrum.01171-23)
Supplement: Supplemental file 3 — Table S3. Download spectrum.01171-23-s0003.docx, DOCX file, 0.02 MB [file spectrum.01171-23-s0003.docx]

**Supplement Table 3.** Rifampicin indeterminate results: stratified analysis of stool/SR mixture storage conditions experiment (A4).

| Storage conditions | | Including all determinate results | | |  | Including only MTB-positive results above MTB detected, trace | | |
| --- | --- | --- | --- | --- | --- | --- | --- | --- |
|  |  | N | % RIF indeterminate | p-value* |  | N | % RIF indeterminate | p-value ‡ |
| Fridge | 5 hrs | 122 | 11% | 0.46 |  | 86 | 0.0% | **0.009** |
|  | 12 hrs | 122 | 11% |  |  | 86 | 0.0% |  |
|  | 24 hrs | 122 | 16% |  |  | 79 | 5.1% |  |
|  | Total | 366 | 13% |  |  | 251 | 1.6% |  |
| RT | 5 hrs | 122 | 9% | **0.024** |  | 75 | 2.7% | 0.52 |
|  | 12 hrs | 122 | 14% |  |  | 74 | 1.4% |  |
|  | 24 hrs | 122 | 21% |  |  | 65 | 4.6% |  |
|  | Total | 366 | 15% |  |  | 214 | 2.8% |  |
| Overall | | 732 | 14% |  |  | 465 | 2.2% |  |
| 5 hrs | Fridge | 122 | 11% | 0.527 |  | 86 | 0.0% | 0.215 |
|  | RT | 122 | 9% |  |  | 75 | 2.7% |  |
|  | Total | 244 | 10% |  |  | 161 | 1.2% |  |
| 12 hrs | Fridge | 122 | 11% | 0.436 |  | 86 | 0.0% | 0.462 |
|  | RT | 122 | 14% |  |  | 74 | 1.4% |  |
|  | Total | 244 | 12% |  |  | 160 | 0.6% |  |
| 24 hrs | Fridge | 122 | 16% | 0.248 |  | 79 | 5.1% | 1 |
|  | RT | 122 | 21% |  |  | 65 | 4.6% |  |
|  | Total | 244 | 18% |  |  | 144 | 4.9% |  |
| Overall | | 732 | 14% |  |  | 465 | 2.2% |  |
| Combi-nation of tempe-rature and time | Fridge 5H | 122 | 11% | 0.072 |  | 86 | 0.0% | 0.053 |
|  | Fridge 12H | 122 | 11% |  |  | 86 | 0.0% |  |
|  | Fridge 24H | 122 | 16% |  |  | 79 | 5.1% |  |
|  | RT 5H | 122 | 9% |  |  | 75 | 2.7% |  |
|  | RT 12H | 122 | 14% |  |  | 74 | 1.4% |  |
|  | RT 24H | 122 | 21% |  |  | 65 | 4.6% |  |
|  | Total | 732 | 14% |  |  | 465 | 2.2% |  |

* Chi-square; ‡ Fisher’s exact.
