# Supplementary material for: Optimization of the Simple One-Step Stool Processing Method to Diagnose Tuberculosis: Evaluation of Robustness and Stool Transport Conditions for Global Implementation
Source: Microbiol Spectr. 2023 Jun 26;11(4):e01171-23. doi: 10.1128/spectrum.01171-23 (PMC10434014; doi:10.1128/spectrum.01171-23)
Supplement: Supplemental file 4 — Figure S1. Download spectrum.01171-23-s0004.docx, DOCX file, 0.6 MB [file spectrum.01171-23-s0004.docx]

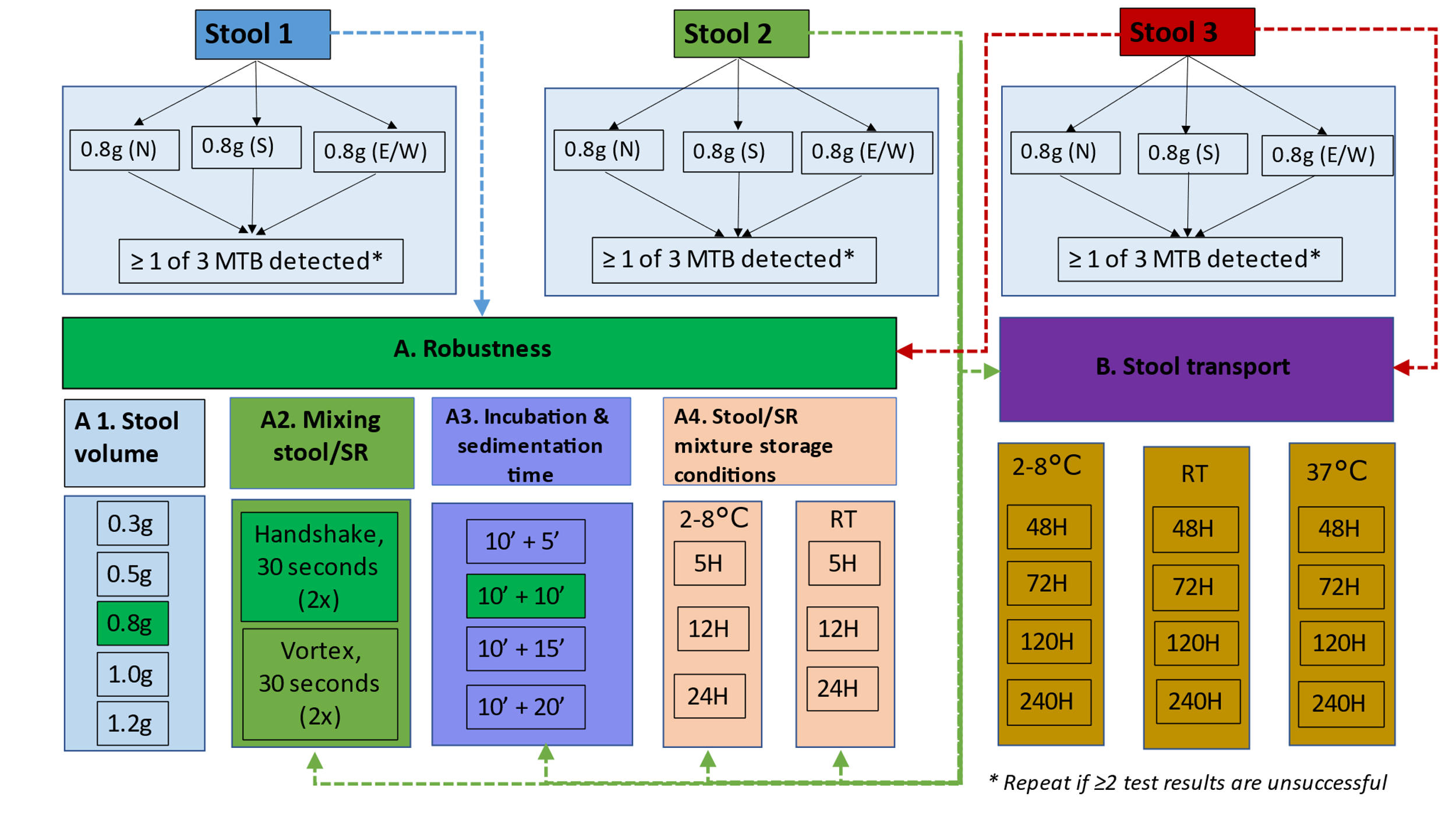


**Supplement Figure 1.** Overview of Experiments: The arrows indicate experiments that were carried out using various stool specimens. Abbreviations used: E/W, East or West; Exp, experiment; g, gram; H, hours; MTB, *M. tuberculosis*; N, North; RT, room temperature; SR, sample reagent; S, South.
