# Supplementary material for: Optimization of the Simple One-Step Stool Processing Method to Diagnose Tuberculosis: Evaluation of Robustness and Stool Transport Conditions for Global Implementation
Source: Microbiol Spectr. 2023 Jun 26;11(4):e01171-23. doi: 10.1128/spectrum.01171-23 (PMC10434014; doi:10.1128/spectrum.01171-23)
Supplement: Supplemental file 5 — Figure S2. Download spectrum.01171-23-s0005.docx, DOCX file, 0.02 MB [file spectrum.01171-23-s0005.docx]

No data

No data

**Supplement Figure 2.** Association between stool volume and risk of processing errors, relative to adding 0.8g of stool as the reference standard. There were no invalid results at 0.3g and 1.2g.
